# Severe liver injury affects the outcomes and length of hospital stay in children with community-acquired pneumonia

**DOI:** 10.4314/ahs.v22i3.62

**Published:** 2022-09

**Authors:** Lianyu Zhang, Shuai Zhao

**Affiliations:** 1 Division of Neonatology, Department of Pediatrics, the Affiliated Hospital of Southwest Medical University, Luzhou, Sichuan, China; 2 Sichuan Clinical Research Center for Birth Defects

**Keywords:** Community-acquired pneumonia, liver injury, children, mortality, hospital length of stay

## Abstract

**Background:**

The present study was undertaken to evaluate the association between liver injury and clinical parameters, outcomes and length of stay (LOS) in hospital in children with community-acquired pneumonia (CAP).

**Methods:**

Clinical data and laboratory indicators of 2,573 children with CAP were analyzed. The association between liver injury and clinical parameters, outcomes and LOS was then analyzed.

**Results:**

Higher liver injury class was associated with higher incidence of severe CAP, comorbidities, hypoxia, requirement for mechanical ventilation, 30-day mortality and intensive care unit admission, and higher indicators of inflammation (C-reactive protein, procalcitonin and white blood cell count), longer LOS, faster respiratory rate and pulse rate, and lower age, serum albumin levels, monocyte and lymphocyte counts. Severe liver injury was identified as an independent factor for 30-day mortality and prolonged LOS in children with CAP. Higher liver injury class was associated with a lower cumulative survival rate (p=0.0004), and log-rank test for trend was used to demonstrate the association of each injury class with 30-day mortality (p=0.0002).

**Conclusions:**

Several parameters were associated with liver injury in children with CAP. Severe liver injury was found to be an independent factor for 30-day mortality and LOS in children with CAP.

## Introduction

It has been demonstrated that pneumonia affects 50% of children annually in the least developed countries or regions 1 and it is the most common reason for childhood hospitalization, even in countries with a higher socioeconomic status, such as the United States of America (USA). The medical cost of pediatric pneumonia in the USA was estimated at almost $1 billion in 2009 2. In China, there are an estimated 21.1 million new cases of pneumonia annually in children aged <5 years 3. Pneumonia is also a leading cause of hospitalization among children, with infants and young children being particularly susceptible, and is the second leading cause of mortality among children aged <5 years in China 4. Pneumonia is the leading cause of death in children aged <5 years worldwide, with nearly 2 million deaths, representing 19.0% of total deaths in children in 2010 5, and has long been recognized as a major health concern worldwide. Community-acquired pneumonia (CAP) is the predominant cause of childhood morbidity and mortality in the world, causing nearly 1.2 million deaths annually among children aged <5 years 6.

CAP caused by different pathogens usually affects extrapulmonary organs, as a number of studies have reported that acute kidney injury in adults with CAP was associated with higher mortality and the patient was more likely to require mechanical ventilation 7–9, and similar studies also have been found in children with CAP 10, 11. Moreover, myocardial injury caused by CAP has been widely investigated 12, 13. These organ injuries could increase the hospitalization rate, socioeconomic burden and mortality rate. The frequency of liver injury in critical illness has been significantly increasing over the last decades, and liver injury is an important predictor of worse outcome and prolonged hospitalization in adults 14. It was previously demonstrated that one-third of pneumonia cases caused by corona virus disease-19 were associated with liver function test abnormalities 15, which was in accordance with the findings reported by Cai et al 16. In addition, Huang et al reported that 13.8% cases of CAP in adults were associated with liver injury 17. Additionally, infections in patients with acute liver injury were associated with increased risk of death or transplant and length of stay (LOS) in hospital 18. However, liver injury and its clinical characteristics in children with CAP have yet to be comprehensively investigated. Hence, the aim of the present study was to report the clinical course, outcomes and liver injury in children with CAP.

## Materials and methods

### Patients

Between January 2010 and March 2015, a total of 2,573 children with CAP who were hospitalized at the Department of Pediatrics of the Affiliated Hospital of Southwest Medical University were enrolled in the present study. All clinicians were trained in order to enroll the eligible children. Children with CAP aged 3 months-14 years were selected according to protocol definitions and inclusion criteria. CAP cases were defined using the Child CAP Guidelines (I, II) of the Chinese Medical Association 19, 20 as follows: i) Recent fever (>37.5°C), cough and/or dyspnea, tachypnea and sputum; ii) fixed moderate or fine/span>rales or dry rales during inspiration detected on lung auscultation; and iii) radiological confirmation of pneumonia, defined as the presence of consolidation (dense or fluffy opacity, with or without air bronchogram), other infiltrates (linear and patchy alveolar or interstitial densities), or pleural effusion within ≤72 h of admission. All radiologists were blinded to the demographic and clinical information of each case. CAP was divided as into mild and severe according to Child CAP Guidelines of the Chinese Medical Association. Since there are no validated severity criteria for children with CAP, severe CAP was defined as follows: Oxygen saturation <92% on room air, tachypnea, dyspnea, poor general condition, signs of dehydration or refusal to eat, altered mental status, cyanosis, pleural effusion, extrapulmonary complications or inflammatory infiltration of multiple lobes, or involvement of more than 2/3 of the lungs. The CAP treatment strategies were consistent with the aforementioned guidelines.

**Figure 1:**
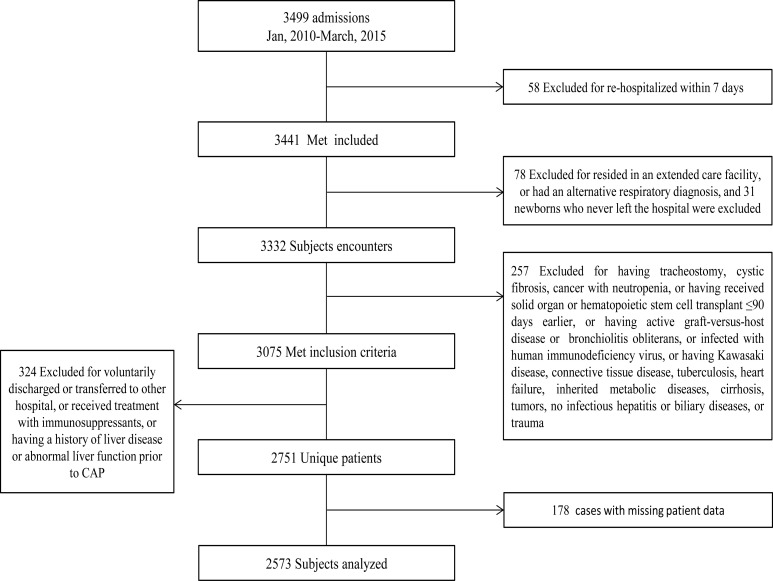
Study flow diagram.

Children were excluded if: i) They were re-hospitalized within 7 days; ii) they resided in an extended care facility, or had an alternative respiratory diagnosis; iii) they were newborns who never left the hospital; iv) they had tracheostomy, cystic fibrosis, cancer with neutropenia, had received solid organ or hematopoietic stem cell transplant ≤90 days earlier, had active graft-versus-host disease or bronchiolitis obliterans, or were infected with human immunodeficiency virus; v) they had Kawasaki disease, connective tissue disease, tuberculosis, heart failure, inherited metabolic diseases, cirrhosis, tumors, no infectious hepatitis or biliary diseases, or trauma; vi) the patients were voluntarily discharged or transferred to other hospital; vii) they received treatment with immunosuppressants; viii) they had a history of liver disease or abnormal liver function prior to CAP; and ix) in cases with missing patient data. The flow sheet research selection was shown in Fig.1.

The study was conducted in accordance with the principles outlined in the Declaration of 1964 Helsinki and its later amendments, and was approved by the Affiliated Hospital of Southwest Medical University Ethics Committee. Written informed consent was obtained from all legal guardians.

### Clinical and laboratory assessments

Patient baseline characteristics, medical history, comorbidities and laboratory results, including white blood cell, lymphocyte, monocyte and platelet counts, serum levels of albumin, hemoglobin, C-reactive protein (CRP), bilirubin, glucose, procalcitonin (PCT), aspartate aminotransferase (AST) and alanine aminotransferase (ALT), as well as ejection fraction, were recorded from the electronic medical database. All measurements were obtained on admission within 2 hours. Patients who had raised ALT or AST >50 U/L were defined as liver injury due to the lack of clear guidance or consensus. Liver injury was classified according to the levels of ALT or AST as mild (≤150 U/L), moderate (≤250 U/L), or severe (>250 U/L).

### Outcomes

All follow-up data were obtained from hospital records. The primary outcome variables of interest were LOS, mechanical ventilation, intensive care unit (ICU) admission and 30-day mortality.

### Statistical analysis

All data were analyzed with SPSS 22.0. All continuous variables are presented as median (interquartile range) for skewed distributions and were compared using the Mann-Whitney U test. Categorical variables were expressed as frequencies and percentages, and were compared using the χ2 test or Fisher's exact test (where any cell contained <10). Cumulative survival curves were generated using the Kaplan-Meier method, and survival data were compared among groups using log-rank test and log-rank test for trend. Univariate and multivariate logistic regression analyses or Cox regression analysis were performed to estimate the prognostic impact of different factors on the patients using the forward stepwise method with an entry criterion of P<0.05 and a removal criterion of P>0.10, as appropriate. A two-tailed P-value <0.05 was considered to indicate statistically significant differences for each analysis.

## Results

### Baseline clinical characteristics of patients

The study population included 2,573 children with CAP with a median age of 21 months, of whom 54.6% (1,406) were male. Overall, 317 (12.3%) patients had severe CAP, 124 (4.8%) required mechanical ventilation, and the 30-day mortality rate was 0.9%. Based on the AST and ALT levels on admission, 1,034 (40.2%) patients had no liver injury. The remaining 59.8% of the patients with liver injury were assigned to injury class as follows: 754 (29.3%) mild, 498 (19.4%) moderate and 287 (11.1%) severe.

Patients with liver injury were more likely to be assigned to the categories with severe CAP and lower age, and were more likely to have comorbidities and hypoxia on admission (Table 1). There were no significant differences between liver injury and no liver injury regarding pre-hospital administration of antiviral, antibiotic and herbal medications (all p>0.05). Higher liver injury class on admission was found to be associated with the indicators of inflammation (CRP, PCT and WBC), faster respiratory rate and pulse rate, lower serum albumin levels, monocyte and lymphocyte counts (Table 2).

**Table 1:** Demographics, comorbidities, medication use, and severity of patients with and without liver injury

Demographic	Normal	Mild	Moderate	Severe	*p* value
No.	1,034	754	498	287	
Age (months)	23 (11–33)	23(11–33)	19(10–28)	16(8–24)	0.025
<12	314 (30.4%)	208 (27.6%)	155 (31.1%)	136 (43.4%)	<0.0001
12–60	579 (56.0%)	476 (63.1%)	257(51.6%)	96 (33.4%)	
>60	141 (13.6%)	70 (9.3%)	86 (17.3%)	55 (19.2%)	
Males, n (%)	589 (57%)	409 (54.2%)	251 (50.4%)	157 (54.7%)	0.116
Comorbidities, n (%)	657 (63.5%)	479 (66.0%)	337 (67.7%)	212 (73.9%)	0.005
Hypoxia, n (%)	35 (3.4%)	26 (3.4%)	74 (14.9%)	58 (20.2%)	<0.0001
Medication use, n (%)					
Prehospital antiviral therapy	781 (75.5%)	552 (73.2%)	356(71.5%)	213 (74.2%)	0.462
Prehospital antibiotic therapy	844 (81.6%)	607 (80.5%)	396 (79.5%)	243 (84.6%)	0.168
Herbal medications	334 (32.3%)	214 (28.4%)	138 (27.7%)	93 (32.4%)	0.141
Severity of pneumonia, n (%)					<0.0001
Severe pneumonia	57 (5.5%)	58 (7.7%)	117 (23.5%)	85 (29.6%)	
Mild pneumonia	977 (94.5%)	696 (92.3%)	381 (76.5%)	202 (70.4%)	

**Table 2:** Clinical features in patients with and without liver injury

Clinical Feature	Normal	Mild	Moderate	Severe	*p* value
No.	1034	754	498	287	
Respiratory rate (breaths/min)	29(27–36)	33(29–38)	36(31–43)	42(35–49)	<0.001
Pulse rate (beats/min)	115(104–127)	117(109–127)	121(111–138)	127(119–146)	0.001
Weight (Kg)	10.9(9.7–12.2)	11.1 (10.2–12.4)	10.1(9.1–11.3)	9.8(8.4–10.5)	0.084
WBC (×10^9^/L)	12.9(10.1–16.4)	14.8(10.9–17.4)	15.6(12.3–17.1)	15.1(12.7–19.5)	0.038
Monocyte (×10^9^/L)	0.7(0.52–1.1)	0.68(0.47–0.83)	0.45(0.21–0.65)	0.33(0.17–0.48)	0.008
Lymphocyte (×10^9^/L)	5.4(2.3–8.2)	5.9(2.4–8.8)	4.5(1.2–7.5)	3.7(0.7–5.9)	0.001
Platelet (×10^9^/L)	268(186–342)	237(133–313)	286(179–358)	254(168–388)	0.3
Hemoglobin (g/L)	112(104–119)	111(104–118)	109(99–117)	110(97–121)	0.47
CRP (mg/L)	8.8(1.4–16.7)	7.8(1.0–14.3)	15.5(5.4–24.9)	17.9(7.8–26.4)	<0.001
PCT (mg/mL)	3.7 (0.9–5.4)	2.8(0.7–4.9)	4.6(1.8–7.6)	5.9(2.6–14.3)	0.002
Bilirubin (mmol/L)	9(6–14)	11(7–16)	12(9–19)	13(8–23)	0.15
Albumin (g/L)	40(36–45)	39(36–45)	37(33–41)	34(28–38)	0.006
Glucose (mmol/L)	5.7(4.5–6.8)	6.0(5.0–7.2)	5.9(4.9–6.7)	6.4(5.6–8.4)	0.2
Ejection fraction (%)	61.2±6.3	61±6.8	60.9±6.6	62±4.8	0.1
ICU admission, n (%)	41(4.0%)	38(5.0%)	89(17.9%)	88 (30.7%)	<0.0001
Mechanical ventilation, n (%)	18(1.7%)	19 (2.5%)	32(6.4%)	55(19.2%)	<0.0001
30-d mortality, n (%)	5 (0.5%)	3(0.4%)	8(1.6%)	8 (2.8%)	0.001
hospital length of stay, d	7(4–11)	8 (5–13)	10(7–14)	14(9–18)	0.002

### Outcomes

The outcomes by variables are summarized in Table 2. Patients with liver injury on admission were more likely to be transferred to the ICU and require mechanical ventilation, and exhibited increased 30-day mortality and LOS. Compared with the normal liver, moderate liver injury was associated with an increased need for mechanical ventilation support to 6.4% (vs. normal liver, 1.7%; p<0.0001), a percentage of ICU admission of 17.9% (vs. normal liver, 4%; p<0.0001), increased 30-day mortality rate to 1.6% (vs. normal liver, 0.5%; p<0.0001). The median LOS for patients with liver injury was 10 days (interquartile range, 7–14 days), vs. 7 days (interquartile range, 4–11 days) for patients with no liver injury (p<0.0001). Higher liver injury class was significantly associated with increased LOS.

### Predicting liver injury

Univariate analysis revealed that lower age, serum albumin levels, and monocyte and lymphocyte counts, higher levels of markers of inflammation (CRP and PCT), presence of comorbidities, hypoxia, severe pneumonia and mechanical ventilation, were all significantly associated with liver injury on admission. In the final multivariable analysis, age (OR=0.924, 95%CI: 0.853–0.989, p=0.035), serum albumin (OR=0.88, 95%CI: 0.788–0.967, p=0.042), lymphocyte count (OR=0.98, 95%CI: 0.96–0.99, p=0.047), PCT (OR=1.208, 95%CI: 1.007–2.512, p=0.03), comorbidities (OR=1.947, 95%CI: 1.023–3.994, p=0.01), hypoxia (OR=2.587, 95%CI: 1.026–5.276, p=0.043), severe pneumonia (OR=2.257, 95%CI: 1.127–5.391, p=0.007) and mechanical ventilation (OR=1.998, 95%CI: 1.322–3.328, p=0.003) were all significant prognostic variables for liver injury in CAP (Table 3).

**Table 3:** Univariate and multivariate analysis of liver injury in CAP

Variables	Univariate analysis			Multivariate analysis		
	OR	95% CI	*p* value	OR	95% CI	*p* value
Age (months)	0.913	0.845–0.986	0.021	0.924	0.853–0.989	0.035
Gender			0.325			
Female	1					
Male	1.008	0.992–1.025				
Comorbidities			0.039			0.01
No	1			1		
Yes	2.16	1.407–4.563		1.947	1.023–3.994	
Hypoxia			0.003			0.043
No	1					
Yes	4.527	1.862–7.201		2.587	1.026–5.276	
Severe pneumonia			0.001	1		0.007
No	1					
Yes	2.759	1.233–6.430		2.157	1.127–5.391	
Mechanical ventilation			0.0001	1		0.003
No	1					
Yes	2.059	1.375–4.328		1.998	1.322–3.328	
WBC (×10^9^/L)	1.005	0.936–1.090	0.815			
Lymphocyte (×10^9^/L)	0.97	0.95–0.99	0.016	0.98	0.96–0.99	0.047
Monocyte (×10^9^/L)	0.89	0.74–0.95	0.01	0.86	0.73–0.94	0.39
Platelet (×10^9^/L)	1	0.992–1.0	0.254			
Hemoglobin (g/L)	1.027	0.991–1.065	0.141			
ESR (mm/h)	0.913	0.845–0.986	0.488			
CRP (mg/L)	2.463	1.078–5.423	0.009	1.977	1.045–3.031	0.085
PCT (mg/mL)	3.404	1.767–7.196	0.002	1.208	1.007–2.512	0.03
Bilirubin (mmol/L)	0.933	0.484–2.937	0.69			
Albumin (g/L)	0.85	0.74–0.89	0.012	0.88	0.788–0.967	0.042

### Predicting LOs

We tested the associations between the related variables and LOS by univariate and multivariate analysis in Table 4. Lower age, serum albumin and hemoglobin levels, and the presence of comorbidities, hypoxia, severe pneumonia, mechanical ventilation, as well as higher liver injury class (moderate and severe), were associated with increased LOS in the univariate analysis. After including all related variables with p<0.05 into a multivariate regression model by the forward stepwise method, it was observed that lower serum albumin levels (OR=0.838, 95%CI: 0.756–0.924, p=0.031) and hemoglobin levels (OR=0.863, 95%CI: 0.8113–0.919, p=0.042), and presence of comorbidities (OR=1.61, 95% CI: 1.112–2.831, p=0.031), severe pneumonia (OR=3.162, 95% CI: 1.15–5.073, p=0.012), mechanical ventilation (OR=3.947, 95% CI: 2.031–7.546, p=0.008) and higher liver injury class (severe) (OR=1.356, 95% CI: 1.108–1.938, p=0.006) were significantly associated with increased risk of prolonged LOS.

**Table 4:** Univariate and multivariate analysis of LOS

Variables	Univariate analysis			Multivariate analysis		
	
	OR	95% CI	*p* value	OR	95% CI	*p* value
Age (months)	0.917	0.837–0.988	0.033	0.9 28	0.846–1.018	0.126
Gender			0.497			
Female	1					
Male	0.832	0.489–1.419				
Comorbidities			0.029			0.031
No	1			1		
Yes	2.685	1.475–5.827		1.6 1	1.112–2.831	
Hypoxia			0.019			0.2
No	1			1		
Yes	1.17	1.036–1.265		1.1 2	0.841–1.205	
Severe pneumonia			0.0001			0.012
No	1			1		
Yes	3.358	1.343–7.509		3.1 62	1.15–5.073	
Mechanical ventilation			<0.0001			0.008
No	1			1		
Yes	4.416	2.165–8.194		3.9 47	2.031–7.546	
Liver injury						
Normal	1			1		
Mild	0.596	0.423–1.003	0.092	0.5 94	0.417–1.001	0.15
Moderate	1.245	1.007–2.548	0.037	1.1 56	0.872–2.196	0.126
Severe	1.659	1.225–2.047	0.001	1.3 56	1.108–1.938	0.006
Lymphocyte (×10^9^/L)	0.956	0.573–2.015	0.691			
Monocyte (×10^9^/L)	0.764	0.418–1.427	0.402			
Hemoglobin (g/L)	0.846	0.795–0.913	0.039	0.8 63	0.8113–0.919	0.042
CRP (mg/L)	1.078	0.789–1.853	0.12			
Albumin (g/L)	0.821	0.749–0.917	0.024	0.8 38	0.756–0.924	0.031

### Predicting 30-day mortality

**Figure 2:**
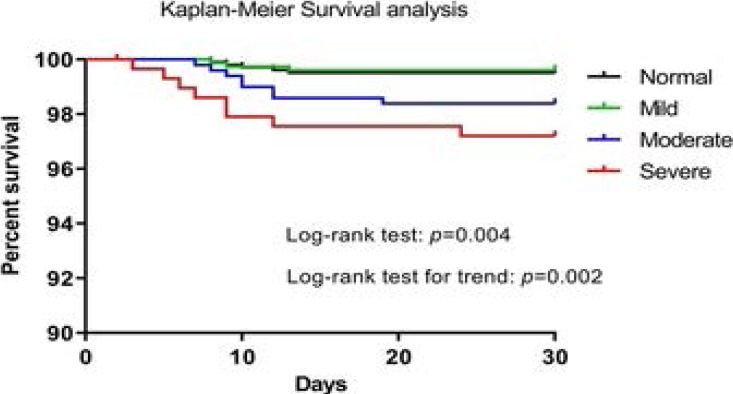
Kaplan-Meier survival curves for 30-day mortality according to the severe categories of liver injury. A gradual increased in 30-day mortality was shown among different injury class

The 30-day Kaplan-Meier survival rates for normal liver, and for mild, moderate and severe liver injury were 99.5, 99.6, 98.4 and 97.2%, respectively. Higher liver injury class had a lower cumulative survival rate (p=0.0004), and log-rank test for trend demonstrated that all injury classes were significantly associated with 30-day mortality (p=0.0002; Fig. 2). Moreover, univariate and multivariate Cox regression analyses were conducted to examine the effects of several parameters on 30-day mortality. Univariate Cox regression analysis revealed that lower agend serum albumin levels, higher incidence of comorbidities, hypoxia, severe pneumonia, mechanical ventilation, as well as higher injury class (moderate and severe), were associated with increased risk of 30-day mortality (all p<0.05). However, age (HR=0.862, 95% CI: 0.832–0.926, p=0.046), presence of comorbidities (HR=1.007, 95% CI: 1.003–1.015, p=0.027), severe pneumonia (HR=1.759, 95% CI: 1.117–3.420, p=0.003), mechanical ventilation (HR=2.334, 95% CI: 1.307–3.462, p=0.002) and severe liver injury (HR=1.062, 95% CI: 1.02–1.093, p=0.035) on admission were independent risk factors for 30-day mortality in the multivariate Cox regression analysis (Table 5).

**Table 5:** Uni- and multivariate Cox regression analyses for mortality of CAP

Variables	Univariate Cox regression			Multivariate Cox regression		
	
	HR	95% CI	*p* value	HR	95% CI	*p* value
Age (months)	0.878	0.859–0.937	0.003	0.862	0.832–0.926	0.046
Gender			0.15			
Female	1					
Male	0.65	0.386–1.253				
Comorbidities			0.003			0.027
No	1			1		
Yes	1.009	1.001–1.018		1.007	1.003–1.015	
Hypoxia			0.007			0.087
No	1			1		
Yes	1.04	1.008–1.083		1.02	1.004–1.059	
Severe pneumonia			<0.0001			0.003
No	1			1		
Yes	2.43	1.541–4.617		1.759	1.117–3.420	
Mechanical ventilation			<0.0001			0.002
No	1			1		
Yes	3.496	1.597–7.294		2.334	1.307–3.462	
Liver injury						
Normal	1			1		
Mild	1.726	0.763–3.50	0.372			
Moderate	1.076	1.006–1.1510	0.032	1.004	0.796–1.135	0.269
Severe	2.16	1.253–3.582	0.005	1.062	1.02–1.093	0.035
Lymphocyte (×10^9^/L)	1.002	0.98–1.024	0.881			
Monocyte (×10^9^/L)	1.027	0.991–1.065	0.141			
Hemoglobin (g/L)	0.938	0.432–2.046	0.87			
CRP (mg/L)	2.538	0.768–4.428	0.128			
Albumin (g/L)	0.873	0.815–0.939	0.008	0.892	0.824–1.046	0.13

## Discussion

To the best of our knowledge, this is the first study to assess liver injury and its clinical characteristics in children with CAP. The present study demonstrated that 59.8% of children with CAP admitted to the hospital had evidence of liver injury, and that age, serum albumin level, lymphocyte count, PCT, presence of comorbidities, hypoxia, severe pneumonia and mechanical ventilation were significant independent variables associated with liver injury in CAP. Evidence of severe liver injury on admission was independently associated with increased 30-day mortality rate and LOS in hospital. To the best of the authors' knowledge, this study is the first to describe the association of increase in LOS and mortality with liver injury on admission for children with CAP.

The frequency of acquired liver injury in critical illness has been significantly increasing over the last decades^14^. In the present study, 59.8% of children with CAP who were admitted to the hospital had different degrees of liver injury. Liver injury has been associated with higher levels of inflammatory makers 21 and hypoxemia 22–24. Hypoxemia was also associated with liver injury on admission in the present study, which was similar to the results reported by Cai et al 16, whereas associations between inflammatory biomarkers (WBC, CRP and PCT) and liver injury were also observed. Additionally, low monocyte and lymphocyte counts may lead to the development of systemic inflammatory response syndrome. Low monocyte and lymphocyte counts were found in CAP with liver injury on admission in the present study, which was in accordance with the findings Huang et al in adult CAP 17. This finding may be attributed to the recruitment of various inflammatory and immune cells to the liver during liver inflammation, with ensuing hepatocyte and biliary epithelial cell injury 25, 26. In addition, age, presence of comorbidities, mechanical ventilation and albumin levels were found to be associated with liver injury in the present study.

It was previously reported that age, comorbidities, hypoxia, severe pneumonia and mechanical ventilation were associated with in-hospital mortality from pneumonia 27, which was in accordance with our findings. The liver is not only susceptible to failure following severe non-hepatic-related insults, but may also aggravate the course of life-threatening conditions. Liver injury has been found to be associated with significantly increased mortality among adults hospitalized in the ICU 14. The present study first demonstrated an increase in 30-day mortality rate among pediatric CAP patients with liver injury, and that higher liver injury class was associated with increased 30-day mortality rate on Kaplan-Meier survival analysis. In the multivariate Cox regression analysis, severe liver injury was an independent predictor of 30-day mortality. Of note, moderate liver injury according to the risk classification was associated with increased need for ICU admission, mechanical ventilation and increased 30-day mortality rate compared with patients in the normal liver group. Alexander et al found that infections in patients with acute liver injury were associated with increased risk of death or transplant 18, further supporting that this cohort of patients requires prompt treatment and close monitoring to prevent deterioration.

Our results indicated that higher liver injury class was associated with increased LOS, and severe liver injury was an independent variable exhibiting a significant positive association with LOS in the multivariate logistic regression analysis. Consistently, LOS was obviously increased among adult patients who had acute liver dysfunction with pneumonia 18; hepatic dysfunction significantly increased LOS, regardless of the presence or absence of other organ dysfunction 28; compared with isolated hepatic injury, patients with concomitant hepatic injury had increased LOS 29. Previous findings have indicated that the predictors of longer hospitalization among children with CAP include presence of comorbidities, severe pneumonia and mechanical ventilation 27, which were consistent with the results of the multivariate regression analysis in this study. Interestingly, these predictors were also associated with liver injury in the present study, to a certain extent, which supported the association between liver injury class and increased LOS. On the other hand, hemoglobin and albumin levels exhibited a significant negative association with LOS in the present study, which were consistent with the findings of previous investigations^30^–^32^. Although there was nossociation between hemoglobin levels and liver injury, a negative association between albumin levels and liver injury was found in this study. A lower serum hemoglobin level reflects a lower capacity for oxygen transport and weakened immunity, which may aggravate infection 33, 34, whereas hypoalbuminemic malnutrition may suppress the activity of T lymphocytes^35^, which may aggravate liver injury and prolong LOS in CAP. However, more studies must be conducted to validate the present results.

There were certain limitations to the present study. First, other indices of liver function, such as gamma-glutamyl-transferase, total bilirubin and alkaline phosphatase, were not analyzed. Second, we did not control for several confounding factors, including other organ injury, complications and possible selection bias. Third, the clinical effects of treatment, including antibacterial and antiviral treatment were unclear. Finally, the pathogens causing CAP and the factors leading to liver injury were not taken into consideration.

## Conclusion

Liver injury in children with CAP was found to be associated with several clinical indicators in the present study, and severe liver injury was identified as an independent factor for 30-day mortality and prolonged LOS in children with CAP.
